# Recovery of cardiac function in Desmin cardiomyopathy with medical therapy

**DOI:** 10.1186/s13023-026-04374-7

**Published:** 2026-04-30

**Authors:** Mengdi Yu, Lutong Pu, Jie Wang, Yucheng Chen

**Affiliations:** 1 Department of Cardiology, West China Hospital, Sichuan University, Guoxue Alley No. 37, Chengdu, Sichuan 610041 China; 2 Cardiac Imaging and Target Therapy Lab, West China Hospital, Sichuan University, Chengdu, Sichuan 610041 China

**Keywords:** Desmin, *CRYAB*, Cardiomyopathy, Cardiovascular magnetic resonance, Heart failure

## Abstract

**Background:**

Desmin cardiomyopathy is a rare hereditary cardiomyopathy caused by *DES* gene variants, often presenting with a spectrum of phenotypes and frequently associated with a poor prognosis.

**Results:**

We report a case of a 24-year-old asymptomatic male referred for abnormal electrocardiogram (ECG) findings. Subsequent investigations revealed worsening left ventricular function and elevated cardiac biomarkers. Diagnosis of Desmin cardiomyopathy was confirmed by cardiovascular magnetic resonance (CMR) showing a characteristic ring-like pattern of late gadolinium enhancement (LGE), endomyocardial biopsy, and genetic testing revealing *DES* and *CRYAB* variants. The patient declined device therapy and was treated with guideline-directed medical therapy (GDMT) in combination with vericiguat. At the 2-month follow-up, the left ventricular ejection fraction was normalized.

**Conclusions:**

Early genetic testing and CMR facilitate diagnosing Desmin cardiomyopathy, and the use of GDMT in combination with vericiguat may lead to significant cardiac recovery.

**Supplementary Information:**

The online version contains supplementary material available at 10.1186/s13023-026-04374-7.

## Introduction

Desmin cardiomyopathy is a rare hereditary cardiomyopathy caused by a variant in the *DES* gene and is pathologically characterized by cytoplasmic desmin-positive immunoreactivity. It encompasses a broad spectrum of phenotypes, including dilated, restrictive, and occasionally hypertrophic or arrhythmogenic cardiomyopathy [1]. Owing to its clinical heterogeneity and rarity, the diagnosis of Desmin cardiomyopathy is frequently delayed or initially missed. The disease is generally associated with a poor prognosis, characterized by progressive heart failure (HF) and a high incidence of malignant arrhythmias [2]. Therefore, cardiac resynchronization therapy with a defibrillator (CRT-D) is commonly recommended. However, we report a unique case of a 24-year-old male with Desmin cardiomyopathy who declined CRT-D but achieved significant myocardial recovery through guideline-directed medical therapy (GDMT) in combination with vericiguat.

## Case presentation

**Fig. 1:**
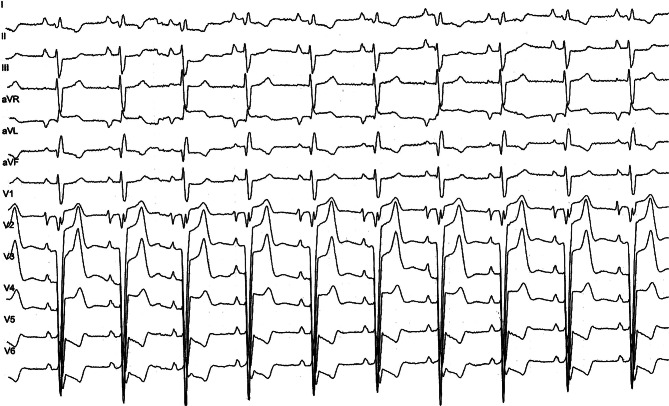
Abnormal 12-lead electrocardiogram (ECG) of a 24-year-old male with Desmin cardiomyopathy. Twelve-lead ECG showed sinus rhythm with first-degree atrioventricular block, intraventricular conduction delay, poor R-wave progression, and ST-segment elevation

A 24-year-old male was referred to cardiology after routine occupational screening revealed electrocardiographic abnormalities. The patient’s father died in his forties from progressive muscle weakness of unknown cause, but detailed clinical records and pathological confirmation were not available. A 12-lead electrocardiography (ECG) showed: (1) sinus rhythm with first-degree atrioventricular block (PR interval 212 ms), (2) intraventricular conduction delay (QRS duration 160 ms), (3) poor R-wave progression in leads V1-V4, and (4) ST-segment elevation (4–7 mm in V1-V3) (Fig. 1). Although asymptomatic (New York Heart Association classification, NYHA functional class I), laboratory tests revealed abnormalities: N-terminal pro-B-type natriuretic peptide (NT-proBNP) 2,690 pg/mL (age-adjusted normal < 125 pg/mL) and cardiac troponin T 130.4 ng/L (normal < 14 ng/L). Transthoracic echocardiography (TTE) showed biatrial enlargement (left atrium 42 mm, right atrium 40 mm), mild concentric left ventricular (LV) hypertrophy (interventricular septum 9 mm; posterior wall thickness 12 mm), with preserved ejection fraction (EF) (64% by Simpson’s biplane method). The patient was initially managed as presumed myocarditis and treated with prednisone.

**Fig. 2:**
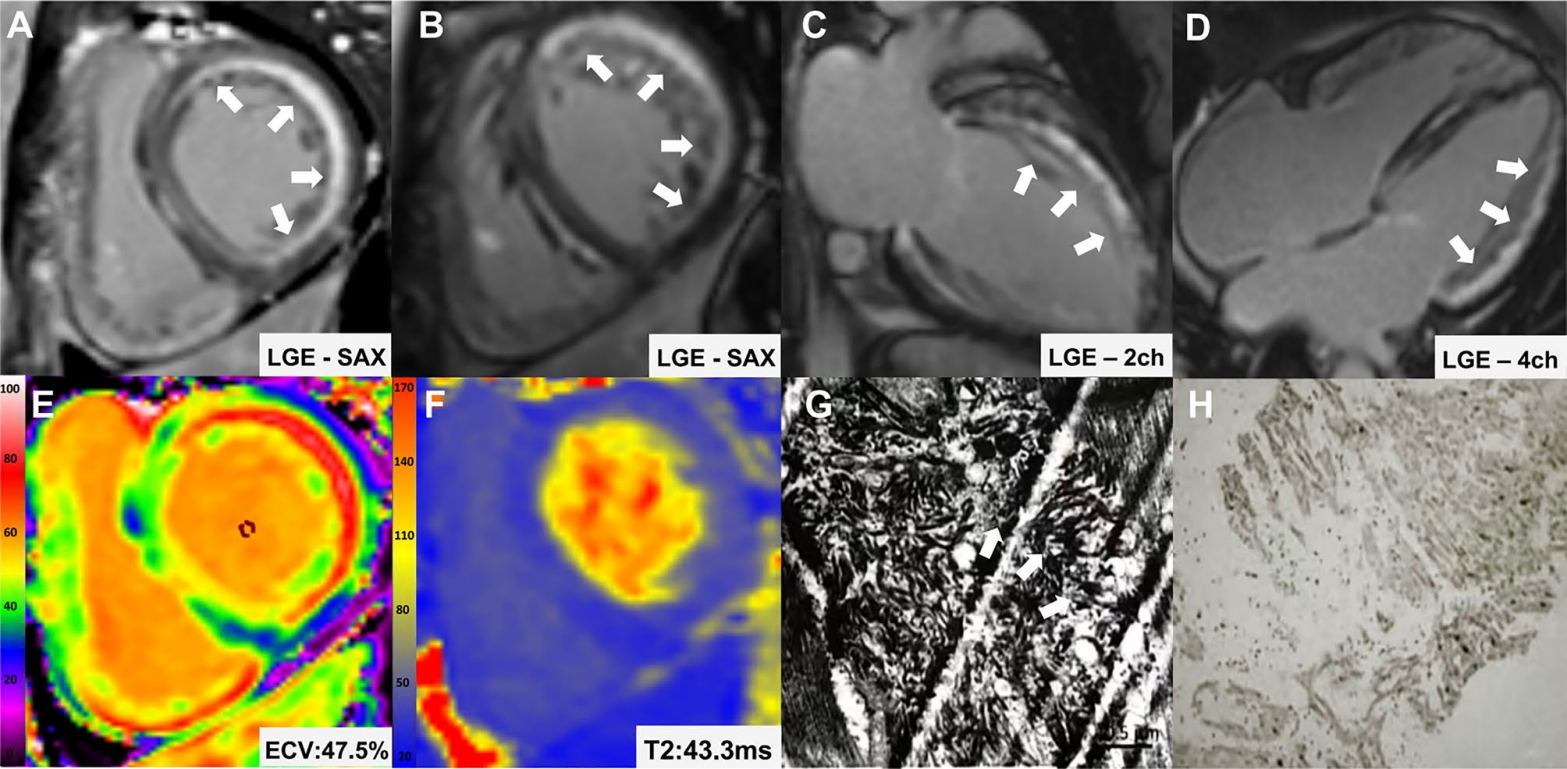
Cardiovascular magnetic resonance (CMR) imaging and endomyocardial biopsy of a 24-year-old male with Desmin cardiomyopathy. CMR imaging demonstrating a characteristic ring-like pattern of late gadolinium enhancement (LGE) in the left ventricular wall (**A**-**D**), elevated extracellular volume (ECV) fraction (**E**), and normal native T2 value (**F**). Endomyocardial biopsy showing abundant autophagic vacuoles and desmin-positive protein aggregates by immunohistochemistry, with corresponding ultrastructural confirmation by electron microscopy (dual staining with uranyl acetate and lead citrate; **G**, **H**). White arrows indicate LGE areas, indicative of myocardial fibrosis. Reference values of ECV: 21.0%-33.0%; reference values of native T2: 40-64 ms. SAX: short-axis view, 2ch: two-chamber view, 4ch: four-chamber view

**Fig. 3:**
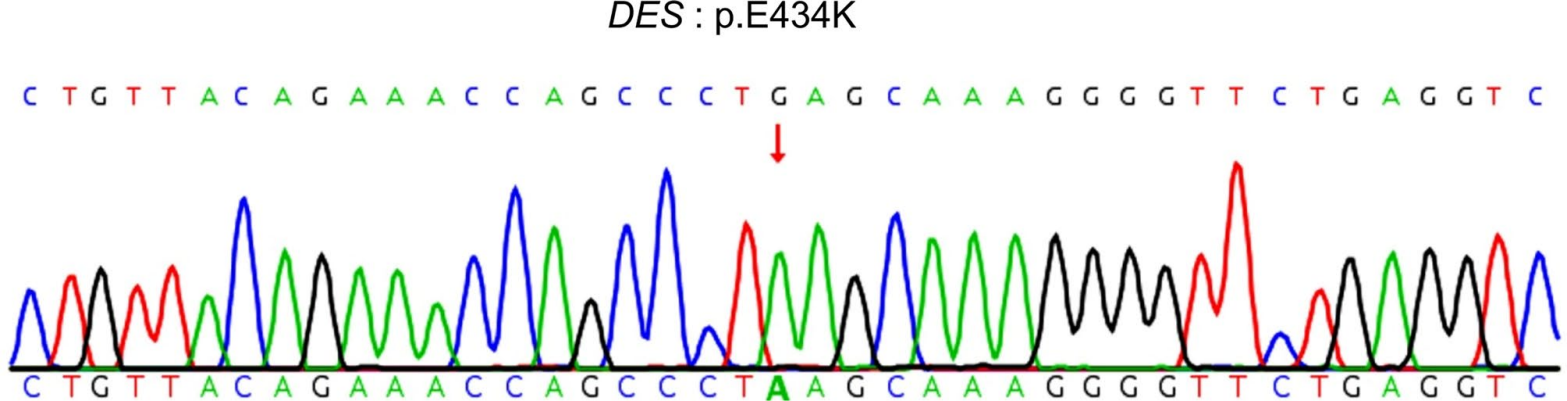
Genotype of a 24-year-old male with Desmin cardiomyopathy. Sanger sequencing showing novel *Des* variant (c.1300G >A)

Seven months later, the patient reported exertional fatigue and dyspnea, corresponding to NYHA functional class II-III. Re-evaluation revealed disease progression with: (1) persistently elevated biomarkers, including NT-proBNP (17,850 pg/mL) and high-sensitivity cardiac troponin I (hs-cTnI) (2.326 ng/mL; normal < 0.034 ng/mL), (2) reduced LVEF to 48%, and (3) mild circumferential pericardial effusion (maximal diastolic separation 8 mm). Given the rapid progression of the disease, the patient underwent further evaluation. 24-hour Holter monitoring revealed frequent multiform premature atrial and ventricular contractions, including episodes of non-sustained atrial and ventricular tachycardia (one episode of each). Cardiovascular magnetic resonance (CMR) revealed: (1) myocardial late gadolinium enhancement (LGE) with a characteristic ring-like pattern (Fig. 2A-D); (2) elevated extracellular volume (Fig. 2E) and normal T2 mapping (Fig. 2F). Endomyocardial biopsy confirmed Desmin cardiomyopathy, including abundant autophagic vacuoles and desmin-positive protein aggregates (Fig. 2G-H). Genetic testing identified likely pathogenic variants: a homozygous *DES* variant (c.1300G > A, p.E434K) (Fig. 3) and a heterozygous *CRYAB* variant (c.4G > A, p.D2N), further confirming the diagnosis. Electromyography examinations revealed no significant abnormalities. The patient was initiated on guideline-directed medical therapy (GDMT), including angiotensin receptor-neprilysin inhibitor (sacubitril/valsartan 50 mg twice daily), beta-blocker (metoprolol succinate 23.75 mg daily), mineralocorticoid receptor antagonist (spironolactone 20 mg daily), and sodium-glucose cotransporter 2 inhibitor (empagliflozin 10 mg daily).

**Fig. 4:**
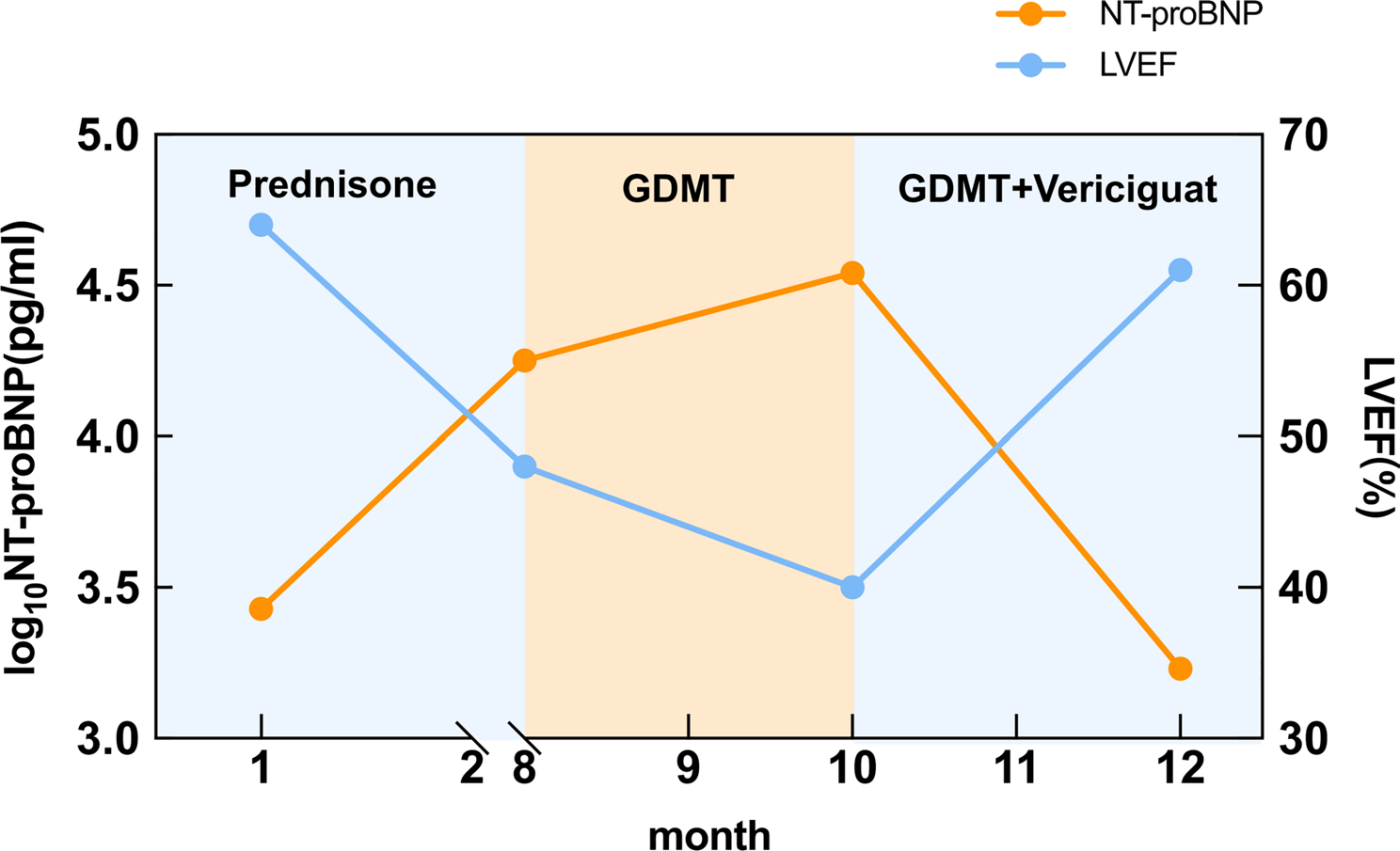
Temporal trends of NT-proBNP and LVEF uring the entire disease course. This figure illustrates the temporal trends in N-terminal pro-B-type natriuretic peptide (NT-proBNP) levels (orange line, left Y-axis) and left ventricular ejection fraction (LVEF; blue line, right Y-axis). As the disease progressed, cardiac function progressively declined. Following the initiation of guideline-directed medical therapy (GDMT) and vericiguat, cardiac function improved, as evidenced by an increase in LVEF and a reduction in NT-proBNP level

Two months after GDMT initiation, the patient experienced clinical deterioration. Symptoms of HF worsened, with dyspnea and fatigue occurring at rest or with minimal exertion (NYHA functional class III). TTE revealed persistent biatrial enlargement (left atrium 44 mm, right atrium 48 mm), moderate tricuspid regurgitation, pulmonary hypertension (estimated systolic pressure 41 mmHg), and a decline in LVEF to 40%. Cardiac biomarkers (NT-proBNP > 35,000 pg/mL; hs-cTnI 17.348 ng/mL). The patient declined cardiac resynchronization therapy with defibrillator (CRT-D) and continued to receive GDMT, with vericiguat initiated and up-titrated from 2.5 mg to 5 mg daily. Remarkably, two months later, he reported substantial relief from dyspnea and fatigue, with improvement to NYHA functional class I-II. LVEF improved to 61% with resolution of pulmonary hypertension and valvular regurgitation, and NT-proBNP decreased to 1,700 pg/mL. At the 8-month follow-up, LVEF remained stable at 62%, with a further reduction of biatrial enlargement (left atrium 34 mm, right atrium 45 mm), and the patient remained asymptomatic (NYHA functional class I), confirming sustained recovery (Fig. 4). The patient’s overall clinical course is summarized in Table 1.

**Table 1:** Clinical Course Summary

Time Point	Symptoms (NYHA)	Laboratory	Echocardiography / Imaging	Treatment / Intervention
Baseline (Initial Presentation)	Asymptomatic (I)	NT-proBNP 2,690 pg/mL; cTnT 130.4 ng/L	Biatrial enlargement (LA 42 mm, RA 40 mm), mild LVH, LVEF 64%	Presumed myocarditis; prednisone
7 Months After Initial Evaluation	Exertional fatigue & dyspnea (II–III)	NT-proBNP 17,850 pg/mL; hs-cTnI 2.326 ng/mL	LVEF 48%; mild pericardial effusion 8 mm	Further evaluation: GDMT initiated (sacubitril/valsartan 50 mg BID, metoprolol 23.75 mg QD, spironolactone 20 mg QD, dapagliflozin 10 mg QD
2 Months After GDMT	Worsening HF, dyspnea at rest or minimal exertion (III)	NT-proBNP > 35,000 pg/mL; hs-cTnI 17.348 ng/mL	Biatrial enlargement (LA 44 mm, RA 48 mm); moderate TR; PH (41 mmHg); LVEF 40%	Patient declined CRT-D; GDMT continued; vericiguat added (2.5 → 5 mg daily)
2 Months After Vericiguat	Substantial relief of dyspnea & fatigue (I–II)	NT-proBNP 1,700 pg/mL	LVEF 61%; resolution of PH and TR	GDMT + vericiguat continued

NYHA: New York Heart Association, NT-pro BNP: N-terminal pro-B-type natriuretic peptide, cTnT: cardiac Troponin T, LA: left atrial, RA: right atrial, LVH: left Ventricular Hypertrophy, EF: ejection fraction, GDMT: guideline-directed medical therapy, QD: once daily, BID: twice daily, HF: heart failure, TR: tricuspid regurgitation, PH: pulmonary hypertension

## Discussion

The *DES* gene encodes desmin, a critical intermediate filament protein of the extrasarcomeric cytoskeleton [3]. Previous studies have reported that in *DES* variant carriers, atrioventricular block occurred in about one-third and ventricular arrhythmias in 5%. Nearly half of carriers developed cardiomyopathy, and overall mortality reached 26% at a mean age of 49 years, with HF and sudden cardiac death being the most frequent causes of death [4]. The *CRYAB* gene encodes αB-crystallin, a chaperone critical for desmin folding and stability. *CRYAB* variants can impair desmin stability and promote its aggregation, leading to cellular dysfunction [5, 6]. Furthermore, *CRYAB* variants disrupt calcium signaling in cardiomyocytes, contributing to pathological hypertrophy [7]. In the present case, the coexistence of a homozygous *DES* p.E434K variant and a heterozygous *CRYAB* p.D2N variant could plausibly contribute to the early onset of cardiomyopathy and rapid progression of HF.

Although the identified *DES* (p.E434K) and *CRYAB* (p.D2N) variants are currently classified as variants of uncertain significance (VUS) under American College of Medical Genetics and Genomics (ACMG) criteria, several points support their potential pathogenic role (Table S1). First, in silico prediction tools (e.g., SIFT, PolyPhen-2, REVEL) suggest deleterious effects of the *DES* variant, while the *CRYAB* variant shows conflicting but partially supportive results. Second, both variants are extremely rare in the East Asian population of the Genome Aggregation Database. Third, the proband’s father had a fatal muscle disorder of unknown cause, raising the possibility of hereditary myopathy. Although segregation analysis could not be performed, the combination of *DES* homozygosity and *CRYAB* heterozygosity may suggest a digenic interaction contributing to the phenotype. This case highlights the importance of genetic testing and reporting rare variants, even when definitive pathogenic classification is not yet possible [8].

On CMR imaging, Desmin cardiomyopathy typically shows a characteristic ring-like pattern of LGE in the LV wall, which is not only useful for identifying specific cardiomyopathy types but also serves as an important indicator of an increased risk of arrhythmias. Parisi et al. recently showed that an LV ring-like LGE pattern on CMR identifies a high-risk phenotype for malignant arrhythmias in patients with non-ischemic cardiomyopathy, with 3.8 events per 100 patient-years [9]. Currently, there is no specific cure for Desmin cardiomyopathy, and conventional pharmacological therapies have limited efficacy. CRT-D, which improves ventricular synchrony and reduces the risk of sudden cardiac death, is commonly considered in high-risk patients [10]. In this case, the patient declined CRT-D and was treated with GDMT combined with vericiguat, resulting in an improvement of LVEF from 40% to 61% within two months.

Vericiguat, as an sGC stimulator, enhances the cyclic guanosine monophosphate–protein kinase G (cGMP–PKG) pathway. Although direct evidence in Desmin cardiomyopathy is lacking, activation of this pathway has been linked to improved cardiac remodeling, mitochondrial regulation, and cytoprotective signaling, which may indirectly modulate autophagy and protein aggregation [11, 12]. This speculative mechanism is consistent with known molecular effects and could partly explain the recovery of cardiac function. The role of vericiguat in advanced HF remains nuanced. In the VICTORIA trial, patients with NT-proBNP > 8,000 pg/mL showed no significant benefit [11]. Despite comprehensive GDMT, our patient experienced rapid deterioration. Careful addition of vericiguat was followed by improved LVEF, resolution of pulmonary hypertension and valvular regurgitation, and marked NT-proBNP reduction. While the exact contribution of each therapy is unclear, this case suggests vericiguat may be considered as an adjunct in progressive HF with reduced EF, warranting further study. Despite the patient’s robust recovery of cardiac function, the presence of ring-like LGE and prior arrhythmias highlights persistent arrhythmic risk, underscoring the need for ongoing surveillance and reassessment of CRT-D candidacy.

This is the first documented case of Desmin cardiomyopathy showing substantial and durable recovery of cardiac function through medical therapy alone. While the exact role of vericiguat is unclear, the patient’s response suggests vericiguat may be an adjunctive option in hereditary cardiomyopathy that continues to progress despite GDMT. Moreover, persistent arrhythmic risk underscores the need for ongoing surveillance and reassessment of device therapy despite preserved LVEF.

## Conclusions

Early genetic testing and CMR play pivotal roles in the timely diagnosis of Desmin cardiomyopathy. The observed substantial cardiac functional recovery following GDMT combined with vericiguat suggests that this therapeutic strategy could be considered in similar patients, although its general efficacy and the exact contribution of vericiguat require validation in larger prospective studies.

## Supplementary Information

Below is the link to the electronic supplementary material.

Supplementary Material 1

## Data Availability

The raw data supporting the conclusions of this article will be made available by the authors without undue reservation.

## Abbreviations

ACMG: American College of Medical Genetics and Genomics
CMR: Cardiovascular magnetic resonance
CRT-D: Cardiac resynchronization therapy with defibrillator
cGMP: Cyclic guanosine monophosphate
ECG: Electrocardiography
ECV: Extracellular volume
EF: Ejection fraction
GDMT: Guideline-directed medical therapy
gnomAD: Genome Aggregation Database
HF: Heart failure
hs-cTnI: High-sensitivity cardiac troponin I
LGE: Late gadolinium enhancement
LV: Left ventricular
LVEF: Left ventricular ejection fraction
NT-proBNP: N-terminal pro-B-type natriuretic peptide
NYHA: New York Heart Association
PKG: Protein kinase G
sGC: Soluble guanylate cyclase
TTE: Transthoracic echocardiography
VUS: Variant of uncertain significance

## References

[1] Papadopoulos C, Malfatti E, Métay C, Keren B, Lejeune E, Buratti J, et al. Deep characterization of a greek patient with desmin-related myofibrillar myopathy and cardiomyopathy. IJMS. 2023 July 6;24(13):11181. 10.3390/ijms. 10.3390/ijms241311181 PMC10342265 37446359

[2] Bermudez-Jimenez FJ, Protonotarios A, García-Hernández S, Pérez Asensio A, Rampazzo A, Zorio E, et al. Phenotype and clinical outcomes in desmin-related arrhythmogenic cardiomyopathy. JACC: Clinical Electrophysiology. 2024 June;10(6):1178–90. 10.1016/j.jacep.2024.02.031. 10.1016/j.jacep.2024.02.031 38727660

[3] Clemen CS, Herrmann H, Strelkov SV, Schröder R, Desminopathies. Pathology and mechanisms. Acta Neuropathol. 2013;125(1):47–75. 10.1007/s00401-012-1057-6. 23143191 10.1007/s00401-012-1057-6 PMC3535371

[4] Van Spaendonck-Zwarts K, Van Hessem L, Jongbloed J, De Walle H, Capetanaki Y, Van Der Kooi A, et al. Desmin-related myopathy. Clin Genet. 2011;80(4):354–66. 10.1111/j.1399-0004.2010.01512.x. 20718792 10.1111/j.1399-0004.2010.01512.x

[5] Wang X, Klevitsky R, Huang W, Glasford J, Li F, Robbins J. αB-Crystallin Modulates Protein Aggregation of Abnormal Desmin. Circul Res. 2003;93(10):998–1005. 10.1161/01.RES.0000102401.77712.ED. 10.1161/01.RES.0000102401.77712.ED 14576194

[6] Thorkelsson A, Chin MT. Role of the alpha-B-crystallin protein in cardiomyopathic disease. Int J Mol Sci. 2024;25(5):2826. 10.3390/ijms25052826. 38474073 10.3390/ijms25052826 PMC10932246

[7] Xu F, Yu H, Liu J, Cheng L. αB-crystallin regulates oxidative stress-induced apoptosis in cardiac H9c2 cells via the PI3K/AKT pathway. Mol Biol Rep. 2013;40(3):2517–26. 10.1007/s11033-012-2332-2. 23212619 10.1007/s11033-012-2332-2

[8] Wang J, Russ D, Yang Y, Pu L, Yu M, Zhang J, et al. Genetic architecture of hypertrophic cardiomyopathy in individuals of Chinese and United Kingdom ancestry. Precision Clinical Medicine. 2025 June 28;8(3):pbaf019. 10.1093/pcmedi/pbaf019. 10.1093/pcmedi/pbaf019 PMC12375896 40860236

[9] Parisi V, Graziosi M, Lopes LR, De Luca A, Pasquale F, Tini G, et al. Arrhythmic risk stratification in patients with left ventricular ring-like scar. Eur J Prev Cardiol. 2024;zwae353. 10.1093/eurjpc/zwae353. 10.1093/eurjpc/zwae353 39486037

[10] Maddox TM, Januzzi JL, Allen LA, Breathett K, Butler J, Davis LL, et al. 2021 Update to the 2017 ACC Expert Consensus Decision Pathway for Optimization of Heart Failure Treatment: Answers to 10 Pivotal Issues About Heart Failure With Reduced Ejection Fraction. J Am Coll Cardiol. 2021;77(6):772–810. 10.1016/j.jacc.2020.11.022. 33446410 10.1016/j.jacc.2020.11.022

[11] Chen W, Wu Y, Li W, Song M, Xu K, Wu M, et al. Vericiguat improves cardiac remodelling and function in rats with doxorubicin-induced cardiomyopathy. ESC Heart Fail. 2025 June;12(3):1807–17. 10.1002/ehf2.15186. 10.1002/ehf2.15186 PMC12055379 39822085

[12] Zhu G, Ueda K, Hashimoto M, Zhang M, Sasaki M, Kariya T, et al. The mitochondrial regulator PGC1α is induced by cGMP–PKG signaling and mediates the protective effects of phosphodiesterase 5 inhibition in heart failure. FEBS Lett. 2022;596(1):17–28. 10.1002/1873-3468.14228. 34778969 10.1002/1873-3468.14228 PMC9199229

