# Supplementary material for: Recovery of cardiac function in Desmin cardiomyopathy with medical therapy
Source: Orphanet J Rare Dis. 2026 Apr 30;21:235. doi: 10.1186/s13023-026-04374-7 (PMC13317310; doi:10.1186/s13023-026-04374-7)
Supplement: Supplementary file 1 — Supplementary Material 1 [file 13023_2026_4374_MOESM1_ESM.docx]

**Table S1** ACMG Evidence for Gene Variants

| Gene / Variant | ACMG Code | Evidence Type | Supporting Data | Strength |
| --- | --- | --- | --- | --- |
| *DES* (NM_001927.4):c.1300G>A, p.Glu434Lys (E434K), homozygous | PM2 | Population frequency | Extremely rare in gnomAD exomes (AF = 0.0000014; absent in genomes); no homozygotes reported | Moderate |
|  | PP3 | In silico / conservation | SIFT: deleterious (0); PolyPhen-2: benign (0.369); phyloP100 = 6.366 (conserved) | Supporting |
|  | PP4 | Phenotype specificity | Patient with conduction disease, cardiomyopathy, desmin-positive aggregates (consistent with DES-related MFM1) | Supporting |
|  | — | ClinVar status | VUS (RCV000795953) | — |
| *CRYAB* (NM_001289808.2):c.4G>A, p.Asp2Asn (D2N), heterozygous | PM2 | Population frequency | Extremely rare in gnomAD exomes (AF = 0.0000028; absent in genomes); no homozygotes reported | Moderate |
|  | PP3 | In silico / conservation | Mixed predictions; phyloP100 = 7.675 (highly conserved residue at codon 2) | Supporting |
|  | PP4 | Phenotype specificity | *CRYAB* is highly expressed in the heart; the patient’s family history of muscle disorder (father) is consistent with *CRYAB*-associated disease | Supporting |
|  | — | ClinVar status | VUS (2 submissions) | — |

ACMG: American College of Medical Genetics and Genomics; PM: Pathogenic Moderate evidence; gnomAD:Genome Aggregation Database ; AF：Allele Frequency；PP : Pathogenic Supporting；MFM1: Myofibrillar Myopathy type 1; VUS: Variant of uncertain significance；RCV：Reference ClinVar Variant.
